# Multiple warty dyskeratoma on the scalp^☆^^☆☆^

**DOI:** 10.1016/j.abd.2019.09.015

**Published:** 2019-09-30

**Authors:** Qiang Zhao, Hongmei Zhou, Songmei Geng

**Affiliations:** Department of Dermatology, Second Affiliated Hospital, Xi’an Jiaotong University, Shaanxi Province, China

Dear Editor,

We report a case of multiple warty dyskeratoma (WD). The patient is a 55-year-old Chinese man who presented with a four-year history of multiple pruritic papules and plaques on the scalp. The number and size of the lesions were gradually increasing. No family history of similar lesions was recorded. Physical examination revealed multiple, discrete, hyperkeratotic papules and plaques, but no vesicles or erosions on his scalp (Fig. 1). No other abnormal systemic involvements were found in our case. Biopsy from multiple lesions revealed similar findings, appearing as cup-shaped invaginations filled with keratotic plugs and acantholytic dyskeratotic architecture covered with a fibrotic capsule in the dermis. The invaginations contain numerous clefts and acantholytic dyskeratotic cells located in the lower epidermis (Fig. 2A). Villi lined by a single layer of basaloid cells and typical corps ronds in the thickened granular layer were observed, with moderate inflammatory infiltrate consisting of lymphocytes, histiocytes, and plasma cells in the dermis. It is worth noting that one cyst contains acantholytic dyskeratotic cells in a hair follicle (Fig. 2B). Based on these clinical and histological findings, a diagnosis of multiple WD was made. WD was first described by Szymanski in 1957.^1^ It is a relatively uncommon benign skin condition which frequently arises as a solitary lesion with a central keratotic plug on sun-exposed sites in the older adults. It is usually localized on the scalp, face, or neck, but has occasionally been reported on oral and vulvar mucosae. Multiple WDs are very rare and only six reported cases were retrieved from PubMed. Most were female and two cases had renal dysfunction.^2^ The previously reported lesions were asymptomatic or associated with only mild pruritus. Recently, Xie et al. reported a case with severe pruritus and the infiltration of eosinophilic granulocytes seen on histologic examination.^3^ Microscopically, no eosinophils were observed in our patient, and only mild itching was present. In our male patient, many violaceous, hyperkeratotic plaques on the scalp can be observed, which is different from previously reported cases with papules or nodules. WD had been proposed to originate from the hair follicle or connection to the sebaceous glands. In our patient, cup-shaped invaginations filled with keratotic plugs and acantholytic dyskeratotic cells were located in hair follicles, which supported its association with hair follicle unit. However, there is still not enough evidence to prove its origination from the hair follicle; this is because WD arising on the oral mucosa and subungual area that normally lack hair follicles has been occasionally reported.^4^, ^5^ The most common differential diagnoses of WD include Darier disease, Grover disease, and Hailey-Hailey disease. They are easily differentiated based on clinical and histologic features. Darier disease may be excluded by the lack of typical clinical features such as white and red longitudinal lines in the nail, often terminating in a V-shaped nick, and uncomfortable lesions on the vulva or in the inguinal folds. The primary histological feature of Grover disease is the presence of small foci of acantholysis with dyskeratosis, intraepidermal clefting, and sometimes vesicle formation. Unlike WD, Hailey-Hailey disease usually lacks prominent areas of acantholytic dyskeratosis. Surgical excision is the first-line treatment for an isolated lesion. Tazarotenic acid gel, laser therapy, and other treatments, such as 3% 5-floxuridine, 0.1% tretinoin cream, and calamine lotion have been reported. In the case of our patient, he responded poorly to topical treatment and received a partial resection. Further follow-up is needed.

**Figure 1:**
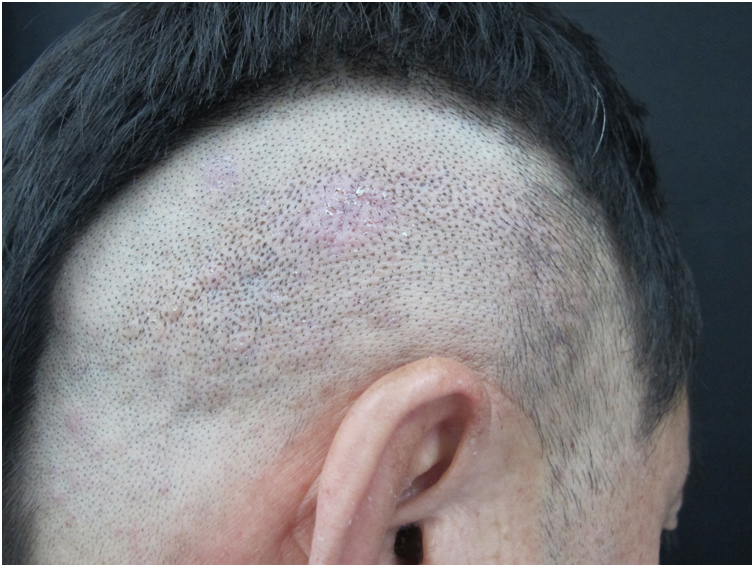
Multiple, firm, violaceous, hyperkeratotic plaques and nodules on the scalp.

**Figure 2:**
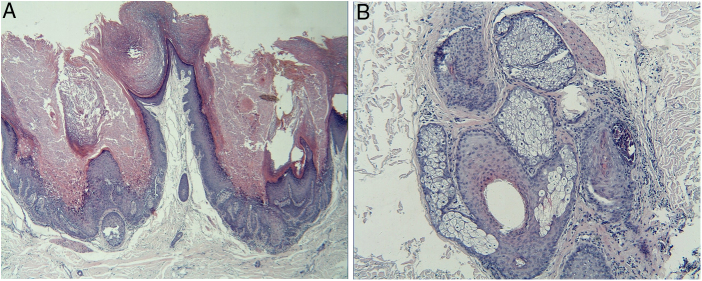
(A) The adjoining cup-shaped invaginations contain numerous clefts and acantholytic dyskeratotic cells located in the lower epidermis (hematoxylin & eosin, ×100). (B) This cyst contains acantholytic dyskeratotic cells in a hair follicle. (hematoxylin & eosin, ×200).

## Financial support

None declared.

## Author's contributions

Qiang Zhao: Elaboration and writing of the manuscript.

Hongmei Zhou: Obtaining, analyzing and interpreting the data.

Songmei Geng: Approval of the final version of the manuscript.

## Conflicts of interest

None declared.

## References

[1] Szymanski F.J. (1957). Warty dyskeratoma: a benign cutaneous tumor resembling Darier's disease microscopically. AMA Arch Derm.

[2] Griffiths T.W., Hashimoto K., Sharata H.H., Ellis C.N. (1997). Multiple warty dyskeratomas of the scalp. Clin Exp Dermatol.

[3] Xie Y., Zhang Q., Wang L. (2018). Multiple warty dyskeratomas with severe pruritus. Am J Dermatopathol.

[4] Peters S.M., Roll K.S., Philipone E.M., Yoon A.J. (2017). Oral warty dyskeratoma of the retromolar trigone: an unusual presentation of a rare lesion. JAAD Case Rep.

[5] Vargas-Laguna E., Imbernón-Moya A., Aguilar-Martínez A., Burgos F. (2017). An unusual location of subungual warty dyskeratoma: a case report and review of the literature. Case Rep Dermatol Med.

